# Synthesis of polypyrrole/cellulose nanocrystals disks for removal of pyocyanin metabolite biomarker released by Pseudomonas aeruginosa

**DOI:** 10.1371/journal.pone.0327713

**Published:** 2025-07-11

**Authors:** Waleed A. El-Said, Ziya A. Khan, Deia A. El-Hady, Wael Alshitari, Mostafa Kamal Masud, Yusuke Yamauchi

**Affiliations:** 1 Department of Chemistry, College of Science, University of Jeddah, Jeddah, Saudi Arabia; 2 Australian Institute for Bioengineering and Nanotechnology (AIBN), The University of Queensland, Brisbane, Queensland, Australia; 3 Department of Materials Process Engineering, Graduate School of Engineering, Nagoya University, Nagoya, Aichi, Japan; 4 Department of Chemical and Biomolecular Engineering, Yonsei University, Seoul, South Korea; VIT University, INDIA

## Abstract

*Pseudomonas aeruginosa* is a high-risk pathogen associated with several human diseases. Pyocyanin (PYO), a redox-active secondary metabolite produced by *P. aeruginosa*, plays a critical role in its survival and pathogenicity, exhibiting both antibacterial and toxic properties. Recent studies have shown that reducing PYO production can inhibit the growth of P. aeruginosa. Here, we report the extraction of cellulose nanocrystals from rice husk for the fabrication of cellulose nanocrystal/polypyrrole (PPy/cellulose) composite disks. This nanocomposite disk acts as a simple, highly efficient, and cost-effective adsorbent for removing PYO metabolites from contaminated water samples. The chemical and morphological features of the PPy/cellulose composites are investigated using various techniques. Solid-phase extraction is employed to remove PYO, with treatment conditions optimized for maximum efficiency. Both two-parameter and three-parameter models are used to analyze the equilibrium data for PYO removal. The optimal adsorbent dose is found to be 20 mg at 303 K for 35 minutes. The PPy/cellulose disk reaches maximum adsorption, removing over 93% of 10 ppm PYO. This approach presents a novel and effective strategy for mitigating the harmful effects of PYO, with potential applications in treating *P. aeruginosa* infections and recycling PYO for antimicrobial use.

## 1. Introduction

*Pseudomonas aeruginosa (P. aeruginosa)* is an invasive microorganism capable of aerobic and anaerobic respiration. It is among the most prevalent human pathogens and can be fatal in critically ill patients, such as those with cancer or premature infants [1–4]. *P. aeruginosa* is widely distributed in water, humans, animals, plants, soil, and sewage. Infections typically occur through wounds or in patients with cystic fibrosis and are characterized by multidrug resistance [5–9], leading to prolonged hospitalization, increased medical costs, and higher mortality [10]. Treating *P. aeruginosa* infections is challenging because of its antibiotic resistance [11]. The pathogenicity of *P. aeruginosa* is partly attributed to the secretion of phenazine metabolites [12,13], which play essential roles in bacterial survival, including biofilm formation and growth. Among these phenazine metabolites, pyocyanin (PYO) is particularly important for bacterial growth and biofilm formation [13,14]. PYO also acts as an oxygen scavenger due to its redox activity [15,16], exhibits strong antibacterial and antifungal properties, inhibits biofilm formation in several bacteria [17,18] and can destroy the host cells by disrupting critical cellular processes, generating reactive oxygen species (ROS), and inducing neutrophil apoptosis [19]. PYO is the primary biomarker for *P. aeruginosa* and is used to monitor disease progression in conditions such as lung infection, corneal perforation, and lung failure [20–32]. As one of several toxins secreted by *P. aeruginosa*, mitigating PYO toxicity has become a significant research focus [33,34]. Thus, avoiding the toxicity of the PYO has potential interest. For example, Nafee et al. reported that solid lipid nanoparticles (NPs) can penetrate the polysaccharide barrier in biofilms and significantly reduce PYO production [22].

Due to its biological importance, several methods have been reported for extracting and purifying PYO [35]. Reducing PYO production has emerged as a promising strategy for managing *P. aeruginosa* infections. Various nanomaterials have been explored for this purpose, including TiO₂ nanoparticles, chitosan-metal oxide nanocomposites, polymer-based nanocomposites, and metal oxide-based nanocomposites [36–39]. Among available strategies, adsorption using naturally abundant, waste-derived, and by-product materials offers significant advantages, such as lower environmental impact and cost-effectiveness, making it particularly appealing for large-scale applications [40–45]. Therefore, there is a growing need to develop sustainable, non-toxic sorbents capable of efficiently adsorbing, sensing, or removing low concentrations of harmful biomolecules like PYO.

To evaluate the efficacy of such materials, various chromatography-based, electrochemical, optical, and spectroscopic techniques have been employed. While some offer high sensitivity, they often require extensive sample preparation, are time-consuming, and can be costly. Given that PYO is a redox-active compound with strong absorbance properties, electrochemical and optical methods have gained traction due to their high sensitivity, selectivity, cost-effectiveness, and rapid, direct measurement capabilities [46–48]. The optical techniques have exclusive advantages, including high sensitivity, high selectivity, no sample preparation, ease of use, and fast measurement.

**Fig 1:**
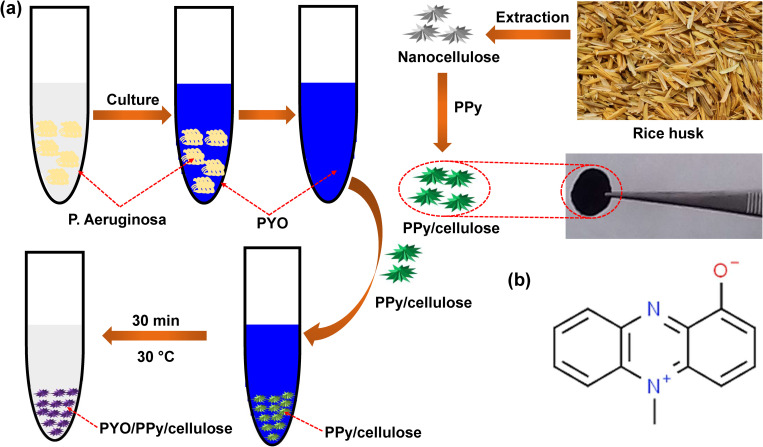
(a) schematic diagram of the preparation of PPy/ cellulose nanocomposite disk and their uses for PYO biomarker removal, and (b) chemical structure of PYO.

In this study, we present a simple and efficient assay for the removal of PYO using PPy/cellulose nanocomposite disks derived from rice husk waste. Cellulose nanocrystals are extracted and incorporated into PPy matrices to form the nanocomposites, which are then characterized and applied for PYO removal from contaminated samples (Fig 1a). Key parameters—such as contact time, treatment temperature, and adsorbent dosage—are optimized. The optimal conditions are found to be 20 mg of adsorbent at 303 K for 35 minutes, achieving over 93% removal of 10 ppm PYO. This novel assay offers a sustainable and effective approach to mitigating the harmful effects of PYO, with potential implications for treating *P. aeruginosa* infections and enabling the recovery of PYO for antimicrobial applications.

## 2. Materials and methods

### 2.1. Chemicals

Pyrrole, ammonium persulfate, PYO (P0046-5MG), and ethanol were bought from Sigma (St. Louis, MO, USA). Lysogeny broth (LB) was purchased from BIO BASIC INC, Markham, Canada. All the chemicals used were of analytical reagent grade.

### 2.2. Instrumentation

X-ray diffraction (XRD) patterns were recorded using a Philips PW 1710 X-ray diffractometer operating at 40 kV and 40 mA, examining particle sizes in the 2θ = 5° – 60° range with nickel-filtered CuKα radiation (λ = 1.54060 Å). Thermogravimetric analysis (TGA) was performed using a Shimadzu TGA 60H instrument. Measurements were conducted with 13 mg of sample under a nitrogen atmosphere (flow rate: 40 mL min^−1^) and a heating rate of 10°C min^−1^. Bruker Senterra Raman microscope (Bruker Optics Inc., Germany) was used to record the PYO Raman spectrum released from *P.* *aeruginosa* bacteria within a Raman shift of 600 − 1800 cm^−1^. The sample was excited with a 785 nm wavelength laser (50 mW power) for an acquisition time of 15 s. UV-vis spectra were achieved using a Thermo Genesys 180 UV–Visible spectrometer within a wavelength range from 220 to 850 nm. Furthermore, the surface area and pore size distribution were evaluated via N_2_ adsorption/desorption using a Micromeritics ASAP 2020 HD88 analyzer. The BET/BJH methods for determining surface area and pore characteristics were performed at −196°C.

### 2.3. *Synthesis of* cellulose *nanocrystal/polypyrrole* (PPy/cellulose) nanocomposite

The PPy/cellulose nanocomposite disks were synthesized using a simple and environmentally friendly method. The process involved the green extraction of cellulose nanocrystals from rice husk, followed by the polymerization of pyrrole and the fabrication of the nanocomposite disks.

Cellulose nanocrystals were obtained from rice husk in two stages, i.e., extraction of cellulose and isolation of the cellulose nanocrystals, as follows. The first stage includes the extraction of cellulose from rice husk using an alkaline-catalyzed condensation reaction. Prior to the alkaline treatment, the rice husks were washed and dried, then waterlogged for a day. Then rice husk was treated and stirred with NaOH 1.8% (w/v) at 100°C for 8 h. Cellulose as a white precipitate was obtained after quenching the solution with an ice bath and collected by filtration. Cellulose was washed several times with deionized water (DI water) and dried at 80°C overnight in an oven. In the second stage, cellulose nanocrystals were isolated from the obtained cellulose using sulfuric acid as follows: Typically, 3 g of cellulose was suspended in 60 mL of sulfuric acid (65%) was heated at 45°C with gentle stirring for 1.5 h. Filter the suspension and wash with DI water several times until a neutral pH is obtained. The obtained material was dialyzed with DI water. The neutralized cellulose nanocrystal suspensions were sonicated for 20 min.

The pyrrole polymerization was carried out using an ammonium persulfate oxidizing agent at 4°C. Pyrrole hydrochloride was prepared in situ based on mixed pyrrole monomer and hydrochloric acid at a ratio (1:1.1, mol: mol) in DI water and stirred for 30 min in an ice bath. Then, 10 mL of ammonium persulfate solution was added dropwise to the reaction mixture. The mix was constantly stirred for a further 4 h at 4°C. The PPy powder was filtered, washed with DI water, and dried at 50°C for 6 h. Finally, the PPy/cellulose nanocrystals disks were prepared by mixing 5% (w:w) of PPy with cellulose nanocrystals; then the disk was designed by evacuating the system under a dynamic vacuum.

### 2.4. *Removal experiment*

The prepared PPy/cellulose nanocomposites have several advantages due to the combined properties of the polymer and the inorganic material, cellulose. The removal process was performed at different contact times (0 min to 40 min), temperatures (298 K to 308 K), and adsorbent doses (2 mg to 70 mg) to optimize the removal performance. A PYO stock solution (5 mmol L^-1^) was prepared in ethanol; then, a series of diluted concentrations within a range from 2 ppm to 150 ppm were prepared in PBS (pH 7). The PYO was treated with the required dose of the nanocomposites, filtered off the composites, and the residual pigment was studied by recording the UV-vis spectrum. The PYO concentrations after treatment were analyzed using a UV spectrophotometer at a wavelength of 690 nm.

### 2.5. Removal of PYO Released from *Pseudomonas Aeruginosa* Cultures

PYO was obtained from *P. aeruginosa* cultures from the Medical Microbiology and Immunology Department (Assiut University) according to the reported protocol [29,30].

## 3. Results and discussion

### 3.1. *Characterizations of* PPy/cellulose *nanocomposite*

**Fig 2:**
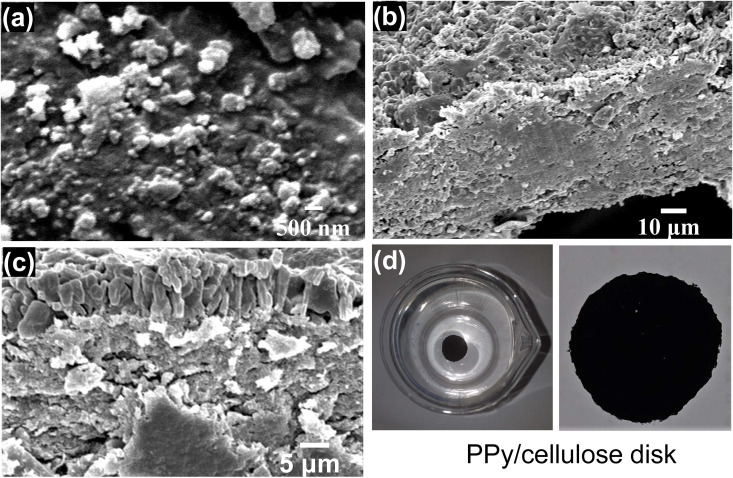
(a) SEM image of cellulose nanocrystals, (b, c) SEM image of PPy/nanocrystals nanocomposites, and (d) photo images of the PPy/cellulose disk.

Cellulose nanocrystals were extracted from rice husk and combined with prepared PPy to develop PPy/cellulose disks. The resulting materials were characterized morphologically and chemically using SEM, XRD, and TGA. For SEM imaging, a small amount of cellulose material was spread as a thin film on a glass substrate and allowed to dry. Fig 2a shows the SEM micrograph of cellulose nanocrystals, revealing spherical nanostructures with an average diameter of 60 nm. In contrast, the morphology of the PPy/cellulose nanocomposites (Fig 2b, c) demonstrates a polymer layer containing embedded cellulose nanoparticles. These results confirm that pyrrole polymerization produces a polymer matrix with a high density of cellulose, making it ideal for PPy/cellulose nanocomposite formation. The photographs of the PPy/cellulose disk was represented in Fig 2d.

**Fig 3:**
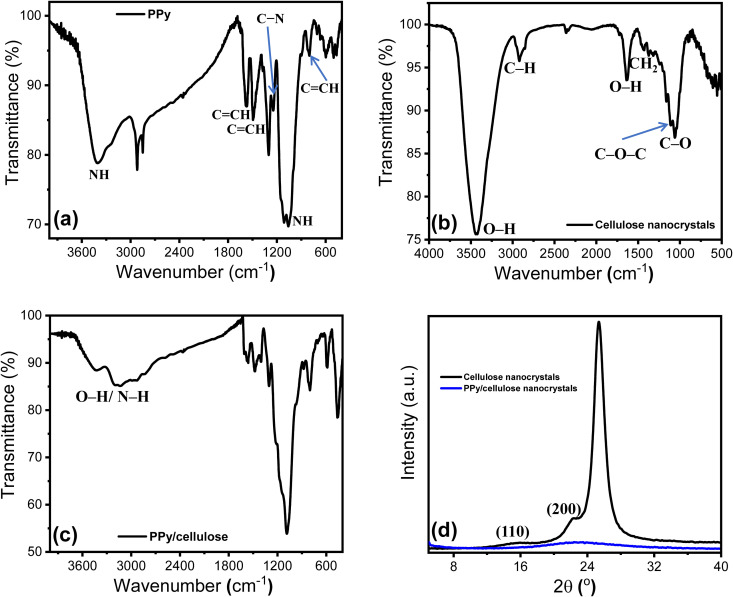
(a) FTIR of PPy, (b) FTIR of cellulose, (c) FTIR of PPy/cellulose disk, and (d) XRD patterns of cellulose nanocrystals and PPy/cellulose disk.

The chemical compositions of PPy, cellulose, and PPy/cellulose composite were investigated using FTIR spectra. Fig 3a shows the FTIR spectrum of PPy, which demonstrates a set of PPy characteristic peaks as follows. The peak at 1244 cm^−1^ is associated with the C − N bond stretching vibration. The peaks at 1559, 1480, and 789 cm^-1^ correspond to the pyrrole ring in- and out-of-plane C = CH vibrations. The vibration peak at 3390 cm^−1^ is related to the N-H group of PPy. In addition, the vibration peak at 1038 cm^−1^ is related to the N–H in-plane deformation of PPy [49].

Furthermore, the FTIR spectrum of cellulose nanocrystals (Fig 3b) shows a spectral peak at 3424 cm^−1^ due to the stretching vibration peak of alcoholic multiple O–H groups in cellulose. Furthermore, the presence of spectral peaks at 2989 cm^−1^ (C–H stretching), 1057 cm^−1^ (C–O bending), 1640 cm^−1^ (O–H bending of adsorbed water), 1420 cm^−1^ (cellulose, CH_2_) and 1117 cm^−1^ (the C–O–C glycosidic linkage) affirmed the cellulose nanocrystals structure [50]. The FTIR of the PPy/cellulose disk (Fig 3c) shows a broad peak at 3412 cm^−1^ that confirms the interaction between the PPy and cellulose via the hydrogen bonding between the N-H (polymer) and OH group (cellulose) [51].

The X-ray diffraction (XRD) technique was used to analyze the crystallinity of the cellulose and PPy/cellulose nanocomposites. The XRD pattern of cellulose (Fig 3d, **black curve**) exhibited a set of peaks at 2θ = 16.5° (110), 22.3° (200), and planes, which are characteristic peaks of cellulose nanostructure [52]. The high intensities of the XRD peaks indicate the high crystallinity of the cellulose. On the other hand, the XRD pattern of the composite (Fig 3d**, blue curve**) showed a significant reduction in the XRD intensity, which confirmed the amorphous phase of the polymer. Fig 3d **(blue curve**) shows the XRD pattern of PPy/cellulose nanocomposites, which illustrates a broad diffraction peak over the 2θ° range from 11.6° to 36.9°. The results confirmed the formation of PPy/cellulose nanocomposite. The broad peak indicates the formation of an amorphous structure due to the presence of a polymer layer.

**Fig 4:**
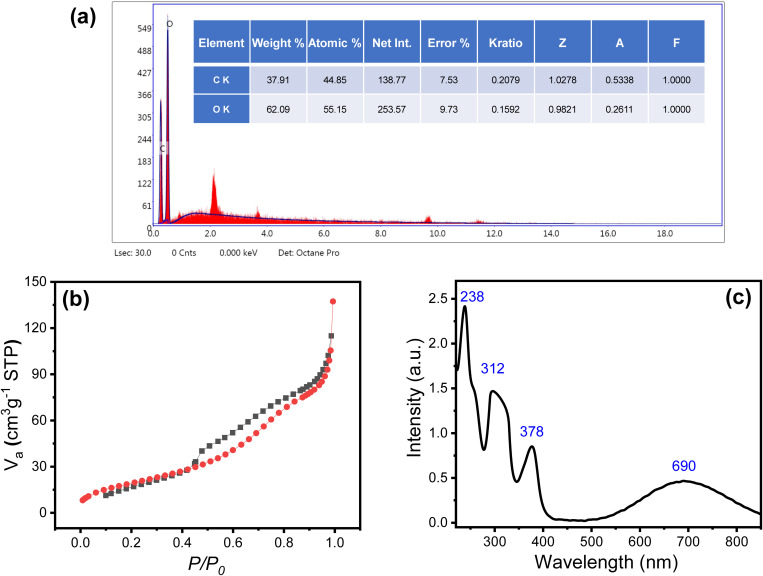
(a) EDX of the cellulose nanocrystals, (b) N_2_ adsorption/desorption isotherm of PPy/cellulose composite, and (c) UV-vis spectrum of 1 mM PYO in PBS (pH 7).

Furthermore, the extracted cellulose was also investigated by using Energy-dispersive X-ray (EDX) technique. Fig 4a shows the EDX spectrum of the cellulose, which shows the distinct and characteristic peaks corresponding to C, and O elements. The results show that the material consists of 37.91% carbon, and 52.09% oxygen. The presence of only C, and O elements confirms the high purity of the extracted cellulose. Moreover, N_2_ adsorption/desorption measurement was performed to study the surface area and porosity of the nanocomposite (Fig 4b). The N_2_ adsorption/desorption isotherm of the composite shows a gradual uptake of nitrogen gas (Fig 4b), which confirms the formation of mesopores in the material. The Brunauer–Emmett–Teller (BET) analysis was applied to calculate the surface area of the composite, which is found to be 101.05 m²g^−1^. Furthermore, N_2_ adsorption/desorption data reveals that the composite nanoparticles have a pore volume of 0.222 cm³.g^−1^ and an average pore diameter of 8.788 nm. These results suggest that the nanocomposite material has a fine, nanoporous structure suitable for applications requiring high surface reactivity or efficient adsorption.

### 3.2. Pyocyanin removal assay

Removal of harmful or precious materials based on the solid phase extraction technique is well reported. Several methods followed the removal efficacy, including chromatography-based, optical, spectroscopic, electrochemical, etc. Here, the UV-vis absorption technique is used to follow the removal efficacy of PYO due to the strong absorption of the PYO pigment, and the unique advantages of optical techniques, including high sensitivity, high selectivity, easy use, and fast measurement.

The PYO removal conditions, including the adsorbent dose, the removal temperature, and the contact time, were investigated to reach the maximum efficacy. pH is another important parameter that affects the removal efficacy. Particularly, compounds like PYO, which contain active functional groups such as hydroxyl and amino groups (Fig 1b). The PYO absorption is based on the medium pH; at neutral pH, PYO exists in its unprotonated form (pKa = 4.9) and shows blue color [53]. Here, the pH was adjusted to 7 during all measurements, which is close to the physiological pH value. Fig 4c shows the UV spectrum of a 1 mM PYO solution in PBS (pH 7), which shows four absorption bands at 238 nm, 312 nm, 378 nm, and 690 nm. These four bands are the specific absorption bands for PYO [30]. The % removal efficacy (RE%) was calculated based on the change of the absorption intensity according to Eq 1. The intensity of the absorption band at 690 nm is used to calculate the RE%. This band is characteristic of the unprotonated form of PYO, which is related to the HOMO–LUMO transition [53].


$$\text{RE}\%\;=\;100\;x\;((I_{0}-I_{f})/I_{0})$$ (1)

Where $I_{0}$ is the initial absorption intensity and $I_{f}$ is the final absorption intensity.

#### 3.2.1. Contact time effect.

**Fig 5:**
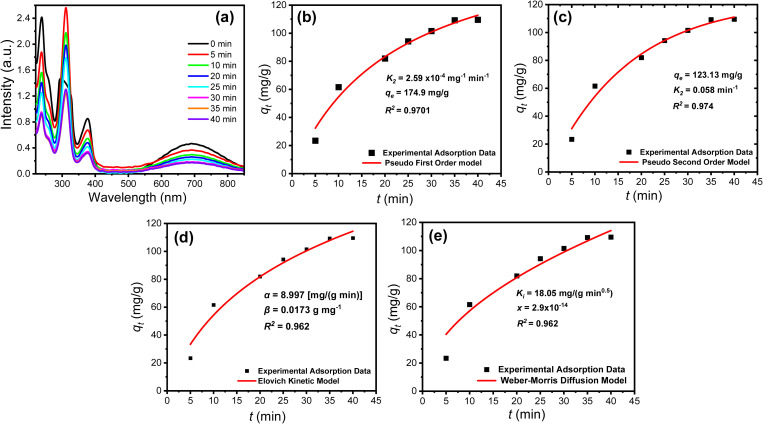
Effect of contact time on the adsorption of PYO biomarker on PPy/cellulose composite from a single solution; initial conc. 120 mg L^-1^, adsorbent wt. 20 mg, solution vol. 20 mL at 303 K: (a) UV-vis spectra of 1 mM PYO in PBS (pH 7) after treatment with 30 mg of PPy/cellulose nanocomposites over different contact times (5-40 min), (b) Pseudo-first order, (c) Pseudo-second order, (d) Elovich, and (e) Weber-Morris diffusion kinetic models.

Contact time plays an essential role in removal efficiency, so the effect of treatment time on removal efficiency was studied. Fig 5a shows the UV-vis spectra of PYO (120 mg L^-1^) before and after being treated with 5 mg of the adsorbent over different periods (0–40 min). The results confirmed that the removal percentage increased with treatment time.

The equilibrium time for adsorption was approximately 35 min, with an initial PYO biomarker concentration of 120 mg L^-1^ at pH 7. Within the first 15 minutes, the uptake efficiency reached 50%, suggesting a moderately paced adsorption process. PYO biomarker capable of diffusing through PPy/cellulose composite pores; requires an extended time to reach equilibrium. The kinetics of the adsorption process are analyzed using various non-linear models, including the pseudo-first order and pseudo-second order equations (Eqs 2 and 3), (Fig 5) [54,55].


$$q_{t}=q_{e}\left(1-e^{-kt}\;\right)$$ (2)

$$q_{t}=\;\frac{k_{2}q_{e}^{2}\;t}{1+k_{2}q_{e}t}$$ (3)

The apparent rate coefficients for pseudo-first-order (*k*_*1*_) and pseudo-second-order (*k*_*2*_) were determined, with *q*_*e*_ representing the adsorption capacity. Plotting *q*_*t*_ versus *t* enabled the estimation of theoretical capacities and rate constants based on the nonlinear curve’s slope and interception. The correlation coefficient value (*R*^*2*^) evaluated the models’ validity, with the pseudo-first-order exhibiting a higher *R*^*2*^ value (0.974) compared to the pseudo-second-order (0.970). This suggests that the first-order model more accurately represents the adsorption rate of PYO biomarker into PPy/cellulose composite. Additionally, the experimental *q*_*e*_ value closely matched the value calculated by the pseudo-first-order model, indicating a physically controlled process. These results suggest progression through stages such as chemical reaction, pore diffusion, film diffusion, and bulk diffusion during the adsorption process.

The Weber-Morris Intra-Particle Diffusion model is applied to further understand the adsorption mechanism and rate-controlling steps influencing the kinetics of PYO biomarker adsorption onto PPy/cellulose composite. According to this model, the diffusion process is radial in direction, and the intra-particle diffusivity is constant (Eq 4).


$$q_{e}=x+K_{i}\;t^{1/2}\;$$ (4)

The nonlinear regression between *q*_*t*_ versus *t* (Fig 5) is utilized to determine the values of *K*_*i*_ (inter-particle diffusion rate constant) and *x* (constant proportional to the thickness of the boundary layer). The high correlation value (0.926) indicates that an intra-particle diffusion mechanism is primarily responsible for PYO biomarker adsorption onto the PPy/cellulose composite. Instead, the low values of *x* (Table 1) suggest that the thinner boundary layer may influence the adsorption process.

**Table 1:** Kinetic Data for the Adsorption of pyocyanin biomarker on PPy/cellulose composite.

Kinetic Models	Parameters	Value
Pseudo first-order model	*q*_*exp*_ (mg g^-1^) *q*_*1*_ (mg g^-1^) *K*_*1*_ (min^-1^) *R*^*2*^	109.2 123.13 0.058 0.974
Pseudo second order model	*q*_*2*_ (mg g^-1^) *K*_*2*_ (mg^-1^ min^-1^) *R*^*2*^	174.97 1.59 x10^-4^ 0.970
Weber-Morris diffusion model	*K*_*i*_ [mg/(g min^0.5^)] *x* *R*^*2*^	18.05 2.9 × 10^-14^ 0.926
Elovich kinetic model	*β* (g mg^-1^) *α* [mg/(g min)] *R*^*2*^	0.0173 8.997 0.962

Another model that we have tested is the Elovich kinetic model, which is usually applicable for situations where the rate-determining step is the interaction between biomarker molecules and the active PPy/cellulose sorbent sites. This model is notable for characterizing the activated chemisorption process and is widely used for chemisorption kinetics. It effectively encompasses a broad spectrum of slow adsorption processes and applies to heterogeneous adsorbent surfaces, as represented by Eq (5).


$$q_{t}=\;\frac{1}{\beta }ln\left(\alpha \beta \right)+\frac{1}{\beta }(t)$$ (5)

where *β* is the adsorption constant (g mg^-1^) and *α* is the initial adsorption rate (mg g^-1^ min^-1^). The deviation of the calculated values of *α* from the experimental *q*_*e*_ is significant, suggesting that the Elovich model is also followed in the adsorption of the PYO biomarker on the PPy/cellulose composite sorbent. The values of α, β, and the respective R^2^ were summarized in Table 1. Thus, the diffusion of PYO in the electric double layer surrounding the adsorbent and in the mesopores regulates the kinetics of the adsorption process (Table 1). The good fitness of the experimental data for Elovich confirmed that a chemisorption process controlled the adsorption of PYO biomarkers onto the PPy/cellulose disk.

#### 3.2.2. Effect of adsorbent doses on the removal of Pyocyanin biomarker.

**Fig 6:**
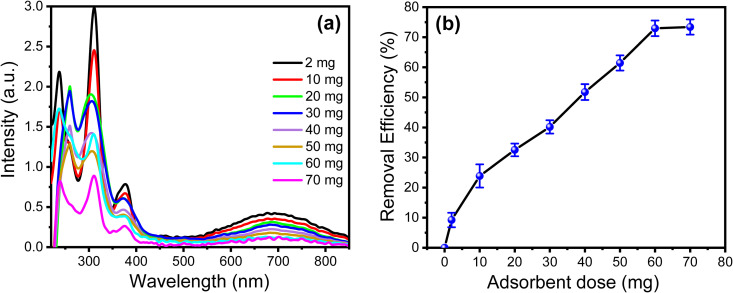
(a) UV-vis spectra of 1 mM PYO in PBS (pH 7) after treatment with several PPy/cellulose nanocomposites adsorbent doses (2 mg – 70 mg) for 35 min at 303 K, (b) effect of PPy/cellulose nanocomposites adsorbent doses on the removal efficiency of PYO.

Several adsorbent doses within a range from 2 mg to 70 mg were used, and their removal efficiency was studied. Fig 6a shows the UV-vis spectra of 5 µM of PYO before and after being treated with different doses of PPy/cellulose adsorbent. The results indicated that the absorption intensity of the PYO solution was reduced after treatment with PPy/cellulose, and the UV-vis intensity was decreased with increasing adsorbent dose. The relationship between the adsorbent dose and the RE% is represented in Fig 6b. The finding indicated that the RE% increased with increasing the adsorbent dose until it reached a steady state at 60 mg of the adsorbent. Furthermore, it confirmed that using 60 mg of PPy/cellulose adsorbent could remove over 73% of the PYO.

#### 3.2.3. Removal capacity of the PPy/cellulose nanocomposite toward the PYO.

**Fig 7:**
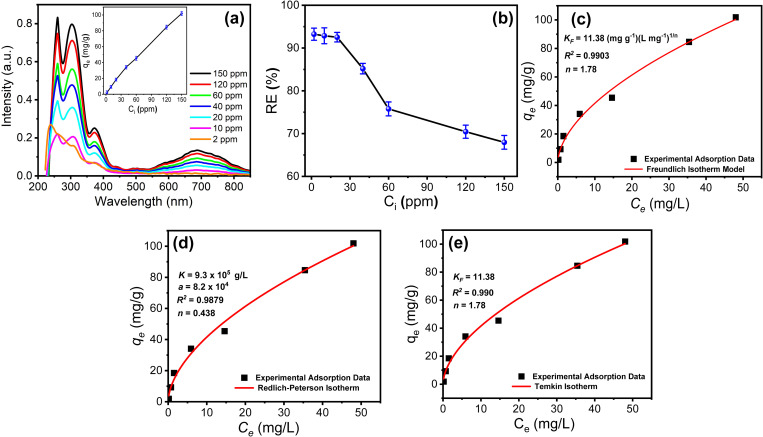
(a) Effect of PYO concentration on the removal efficiency of PPy/cellulose nanocomposite adsorbent, (b) The effects of PYO concentration on the removal efficiency % of the PPy/cellulose nanocomposites adsorbent, (c) Freundlich, (d) Temkin, and (e) Redlich-Peterson models of PYO biomarker (pH 7, eq. time 35 min) on PPy/cellulose from a single ion solution; PPy/cellulose weight 20 mg, solution vol 20 mL.

To examine the removal capacity of the fabricated nanocomposite toward the removal of PYO, the uptake of the composite against different initial concentrations of PYO was studied. A series of PYO concentrations (2 ppm to 150 ppm) was prepared. 20 mL of each solution was treated with 20 mg of the composite at 303 K for 35 min. The adsorbed PYO/nanocomposites were filtered off, and the concentration of the residual PYO was estimated by recording its UV-vis spectrum (Fig 7a). The results indicated that the adsorption capacity (qe) has increased with increasing the initial solution concentration (inset Fig 7a). The initial concentration effect on the removal efficiency is presented in Fig 7b. The results indicated that the removal efficiency was high (over 93%) at lower initial concentrations of PYO. The removal efficiency decreased by increasing the PYO initial concentrations until it reached the saturation state at a PYO initial concentration of 120 ppm with a %R_E_ of about 70%. It is worth mentioning that a few adsorbents were reported for PYO removal. It is reported that 30 mg of macroporous MgO/carbon as an adsorbent removes 90% of PYO [56]. Furthermore, using 10 mg of AST-120 (Kremezin) as an adsorbent for PYO showed 90% removal efficiency [57].

The adsorption isotherms are studied to investigate the adsorbate mass per adsorbent unit weight on the liquid-phase equilibrium concentration of adsorbate and to understand the adsorption equilibrium. Two-parameter models (i.e., Langmuir, Freundlich, and Timken) and the three-parameter model (i.e., Redlich–Peterson, and Sips equations) are investigated to understand the equilibrium data of PYO biomarker removal [58–61]. Using the data collected at pH 7 and 303 K, adsorption isotherms are created to assess the maximum sorption capacity of the PPy/cellulose composite. The PYO biomarker uptake onto PPy/cellulose composite increased gradually, as shown in Fig 7c, and then plateaued at a maximum adsorption capacity of 109.2 mg g^-1^. In addition, the validation of PYO biomarker interaction with PPy/cellulose composite at pH 7 in dilute solutions was affirmed based on sequential monitoring of the UV-vis absorption changes of the solution of very diluted PYO biomarker concentrations (2–150 mg L^-1^). The UV–vis absorbance changes correspond to the PPY/cellulose interaction with the PYO biomarker, and hence, the formation of the [PPy/cellulose → PYO biomarker] complex (Fig 7d). Origin software was used to fit the experimental data with the non-linear forms of the three isotherms, for PYO sorption by PPy/cellulose, and the findings are illustrated in Fig 7. Table 2 shows the Freundlich, Timken, and Redlich–Peterson models’ isotherms constants extracted from the plots in Fig 7. The obtained data illustrated that the Freundlich model [46] showed the best fitting to describe the adsorption equilibrium compared with other models. Determining the Freundlich parameters is directed corresponding to Eq (6):

**Table 2:** Parameters of different models for pyocyanin molecules’ adsorption isotherms.

Isotherm	Parameters	Value
Freundlich	*q*_*exp*_ (mg g^-1^) *K*_*F*_ (mg g^-1^)(L mg^-1^)^1/n^ n *R*^*2*^	102.0 11.38 1.7 0.990
Redlich-Peterson	*A*(L mg^-1^) *β* *B* (L mg^-1^)^1/β^ *R*^*2*^	12.47 0.61 3.02 0.996
Temkin	*b*_*T*_ (KJ mol^-1^) *A*_*T*_ (L g^-1^) *R*^*2*^	3.10 16.5 0.833


$$q_{e}\;=\;K\;C_{eq}^{1/n}$$ (6)

where *n* is the heterogeneity factor, *K*_*F*_ is a Freundlich constant that indicates the relative adsorption capacity of the adsorbent (mg g^-1^), and *q*_*e*_ is the amount of PYO molecules adsorbed per gram of PPy/cellulose (mg g^-1^). An additional isotherm with three parameters is the Redlich-Peterson equation (R-P). This model’s equation is Eq 7.


$$q_{e}\;=\;\frac{AC_{e}}{1\;+\;B\;C_{e}^{\beta }}$$ (7)

The *R-P* constants are namely, *A* and *β*. The equation reduces to a Freundlich isotherm when the value of parameter *B C*_*e*_^*β*^ is much larger than 1, and to a Langmuir isotherm when *β* equals 1. The adsorption capacity is indicated by the *A/B* ratio. The sorption equilibrium data fit well with the Redlich–Peterson model, which combines the characteristics of the Langmuir and Freundlich models. Determination coefficients (R^2^) are determined (0.990) (Table 2), and the values of *b*_*T*_ are not close to unity, thus, the isotherms are approaching the Freundlich form. The Freundlich and Redlich–Peterson models show the highest R^2^ value, so these two models best describe the sorption isotherms of PYO by PPy/cellulose.

The derivation of the Temkin isotherm posits that the decrease in the sorption heat is linear, contrasting with the logarithmic decrease suggested by the Freundlich Eq (8).


$$\text{qe }=\frac{RT}{b_{T}}\text{ln}(A_{T}C_{e})$$ (8)

In this context, *B* = *RT/b*_T_, where *b*_T_ is the Timken constant related to the heat of sorption (J/mol), A is the equilibrium binding constant that represents the maximum binding energy (L/g), *R* is the gas constant (8.314 J/mol K), and *T* is the absolute temperature (K).

The adsorption behavior of PYO onto the PPy/cellulose composite is best described by the Redlich-Peterson (R-P) model (R² = 0.996), which combines features of both Langmuir and Freundlich isotherms. The R-P parameters (A = 12.47 L/mg, β = 0.61) indicate a hybrid adsorption mechanism, where PYO molecules initially form monolayers at high-affinity sites (Langmuir-type) before transitioning to multilayer adsorption (Freundlich-type) on heterogeneous surfaces. This is consistent with the composite π-conjugated polypyrrole backbone, which promotes strong π-π interactions with PYO, while the cellulose substrate provides energetically varied binding sites. The Freundlich model (R² = 0.990, n = 1.7) further supports surface heterogeneity, with n > 1 confirming favorable adsorption. The high KF value (11.38 (mg/g)(L/mg)¹/^n^) suggests significant PYO uptake capacity, attributed to the mesoporous structure and functional groups (–NH, –OH) enabling hydrogen bonding. In contrast, the Temkin model (R² = 0.833) exhibited poorer fit due to its assumption of uniform adsorption energies, which oversimplifies the PPy/cellulose system’s complexity. The low b_T_ value (3.10 kJ/mol) implies weak physisorption, likely dominated by van der Waals forces rather than chemisorption. These findings highlight the importance of hybrid models (e.g., R-P) for accurately describing adsorption on multifunctional composites, where both chemical and physical interactions govern solute uptake. The results align with literature on conductive polymer-based adsorbents, where π-π stacking and hydrogen bonding often synergize to enhance pollutant removal.

#### 3.2.4. Effects of treatment temperature and thermodynamic studies.

**Fig 8:**
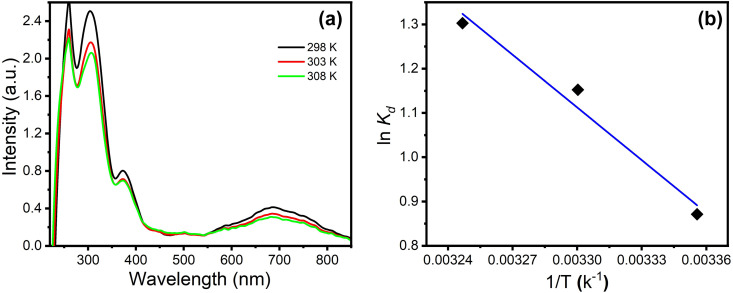
(a) UV-vis spectra of 10 µM PYO in PBS (pH 7) after treatment with 30 mg of PPy/cellulose nanocomposites under different temperatures (298–308 K), and (b) Van ’t Hoff plot of adsorption of PYO molecules on PPy/cellulose composite.


The effect of the treatment temperature (298 K to 308 K) was studied as shown in Fig 8a. 20 mL of each solution was treated with 20 mg of composite from 298 to 303 K for 35 min. The results show that increasing the temperature from 289, 303, and 308 K showed a negligible increase in the removal efficiency (104.8, 114.3, 119.5 mg/g, respectively) which indicated that, the optimal temperature for PYO removal is 298 K. Assessment of the thermodynamic parameters is essential for optimizing the applicability process to demonstrate the practicality of the adsorption process. The thermodynamic parameters of the adsorption of PYO molecules on PPy/cellulose nanocomposites were estimated by substituting *ln K*_*d*_ obtained from the distribution coefficient of the adsorption of PYO on PPy/cellulose nanocomposite at PYO initial concentration of 150 mg L^-1^ at different temperatures of 298, 303, and 308 K into the van ’t Hoff equation, Eq 9.

$${ln\;K}_{d}=\frac{-\Delta H^{\circ }}{RT}+\;\frac{\Delta S^{\circ }}{R}$$ (9)

where *K*_*d*_ is the equilibrium constant and calculated from *K*_*d*_* = q*_*e*_*/C*_*e*_ (L g^-1^) at temperature T. Plotting *lnK*_*d*_ *vs 1/T* of Van’t Hoff equation shows a straight line (Fig 8b) from which the *ΔH°* and *ΔS°* were obtained. The adsorption’s Gibbs free energy (*ΔG°*) was calculated according to Eq 10.


$$\Delta G^{\circ }=-\Delta H^{\circ }+T\Delta S^{\circ }$$ (10)

The values of *∆H*^*◦*^*, ∆S*^*◦*^, and *∆G*^*◦*^ were calculated. The positive value of enthalpy change *∆H*^*◦*^ (33.98 kJ mol^-1^) for the processes affirms the endothermic character. The endothermic nature could be related to either increasing the interaction of PYO molecules with the PPy/cellulose active surfaces or increasing the intraparticle diffusion rate of PYO molecules into the pores at higher temperatures. The positive entropy of adsorption *∆S*^*◦*^ (0.11 J mol^-1^K^-1^) exhibits the adsorbent material’s affinity concerning PYO. Furthermore, the negative free energy values *∆G*^*◦*^ (i.e., 298, 303, and 308 K are −3.2, −2.7, and −2.2 kJ mol^-1^) confirming the possibility and spontaneous nature without an induction period. The inverse relationship between ∆G and temperature indicated the spontaneous sorption of PYO into PPy/cellulose nanocomposite sorbent. The low ∆S value indicates no significant entropy change during the PYO sorption with PPy/cellulose. Furthermore, the positive ∆S value affirms the affinity of the PPy/cellulose sorbent toward PYO and increases the randomness at the solid-solution interface.

### 3.3. *Application* of the adsorbent for the removal of PYO secreted from *Pseudomonas aeruginosa* cultures

**Fig 9:**
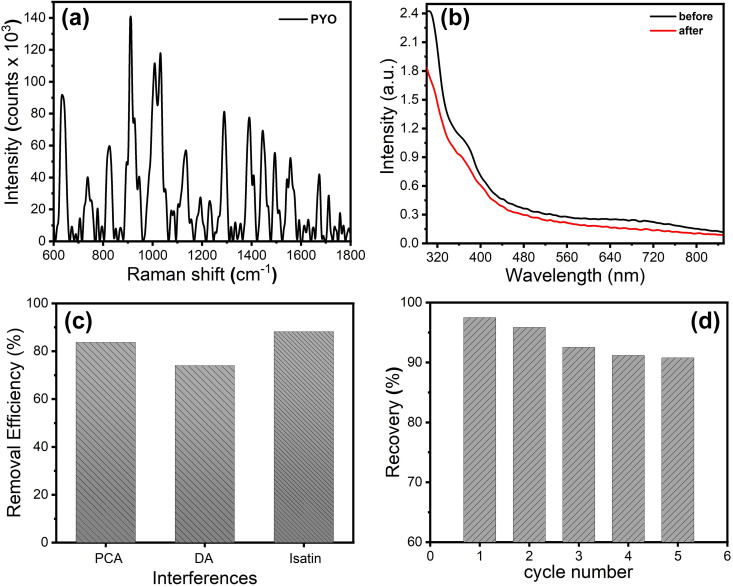
(a) Raman spectrum of PYO released from *P * *Aeruginosa* bacteria, (b) UV-vis spectrum of PYO released from *P.* *Aeruginosa* bacteria before (black curve) and after (red curve) treatment with PPy/cellulose nanocomposites adsorbent, (c) removal efficacy of PPy/cellulose toward PCA, DA & isatin, and (d) recovery of PYO from contaminated samples.

To validate the performance of the prepared nanocomposites as adsorbents for the PYO biomarker, PPy/cellulose nanocomposites were used for PYO removal, which was released from bacteria samples. Although the PYO biomarker is characteristic of its blue color, the presence of PYO in the culture sample was confirmed using Raman spectroscopy before the removal experiment. Fig 9a shows the Raman spectrum of PYO secreted from *P.* *aeruginosa* bacteria, which represents the PYO characteristic peaks, including the Raman peak at Raman shift of 1392 cm^−1^ that is related to the C-C stretching and in-plane C-H bending [31]. Furthermore, the bands at 1560 and 1598 cm^−1^ are ascribed to the ring deformations [31]. Then, the removal experiment of PYO from a real sample was investigated. Fig 9b **(black curve)** shows the UV-vis spectrum of PYO released from bacteria, which illustrates an absorption band at 360 nm besides a broad absorption band over wavelengths from 600 nm to 780 nm. These absorption bands confirmed the presence of PYO in the culture sample. This real sample was treated with 10 mg of PPy/cellulose nanocomposite at 303 K for 35 min and its UV-vis spectrum was Fig 9b **(red curve)**, which showed a decrease in the absorption intensity and hence indicated the capability of the fabricated nanocomposite to remove PYO from a real sample. Furthermore, the removal ability of the PPy/cellulose toward other antimicrobial agents, in addition to some biologically important compounds and organic compounds, was investigated. The following compounds were selected and used as potential interferences: (i) Phenazine-1-carboxylic acid (PCA), one of the phenazines released from Pseudomonas bacteria, (ii) dopamine (DA), one of the neurotransmitters, and (iii) isatin, one of the antitumor drug scaffolds. The results confirm that PPy/cellulose could adsorb these interferences (Fig 9c), which isn’t a critical issue, since most of these interferences are not normally found in the real matrices. Furthermore, the obtained PYO could be purified through different chromatography techniques. Moreover, the future work will focus on minimizing the interference, especially toward the secondary metabolites released from the bacteria.

### 3.4. *Recovery of PYO*

Whereas PYO is a toxin, it has vital biological effects and could be used as a bacterial infection biomarker and as an antimicrobial agent. Hence, the recovery of PYO from contaminated samples has an environmental impact besides its biological roles and the ability to reuse the adsorbent. Here, the recovery of PYO by using PPy/cellulose nanocomposites was investigated. PYO/PPy/cellulose was collected through filtration, and then PYO was recovered using ethanol, and the PPy/cellulose was reused as an adsorbent for 5 cycles. Fig 9d shows the recyclability of the adsorbent and recovery of PYO. The results indicated that the adsorbent was maintained up to the 5^th^ cycle of regeneration/reuse. The recovery efficiency was reduced to 90% after the 5^th^ cycle, which is related to the loss of adsorbent amount during the repeated treatment and filtration.

## 4. Conclusions

In this study, we report the removal of PYO metabolites from bacterial culture media using PPy/cellulose nanocomposites disk. The fabricated nanocomposite disks have been characterized using several analysis techniques, including SEM, TGA, and XRD. The removal efficiency of PYO was monitored by using a UV-vis spectrophotometer. The findings show that the synthesized nanocomposite can potentially remove PYO with a removal efficiency of over 90%. The RE% is found to be dependent on several factors, including adsorbent dosage, contact time, initial concentration, and treatment temperature. The adsorbent also shows excellent stability, maintaining a recovery efficiency of over 90% after five reuse cycles. This work presents a highly efficient approach for the removal of PYO from contaminated water, thereby mitigating its harmful effects.
